# What is the total number of protein molecules per cell volume? A call to rethink some published values

**DOI:** 10.1002/bies.201300066

**Published:** 2013-09-20

**Authors:** Ron Milo

**Affiliations:** 1 Department of Plant Sciences, Weizmann Institute of ScienceIsrael

**Keywords:** cell biology by the numbers, mass spectrometry, protein abundance, proteomic calibration, protein copy numbers, proteins per cell, quantitative proteomics

## Abstract

Novel methods such as mass-spectrometry enable a view of the proteomes of cells in unprecedented detail. Recently, these efforts have culminated in quantitative measurements of the number of copies per cell for most expressed proteins in organisms ranging from bacteria to mammalian cells. Here, we estimate the expected total number of proteins per unit of cell volume using known parameters related to the composition of cells such as the fraction of cell mass that is protein, and the average protein length. Using simple arguments, we estimate a range of 2–4 million proteins per cubic micron (i.e. 1 fL) in bacteria, yeast, and mammalian cells. Interestingly, we find that measured values that are reported for fission yeast and mammalian cells are often about 3–10 times lower. We discuss this apparent discrepancy and how to use the estimate as benchmark to recalibrate proteome-wide quantitative censuses or to revisit assumptions about cell composition.

We estimate the expected total number of proteins per unit cell volume as 2–4 million proteins per cubic micron. Some reported values for fission yeast and mammalian cells using mass spectrometry are 3–10 times lower than these estimates. We discuss this apparent discrepancy and how to recalibrate proteome-wide quantitative censuses.

## Introduction

Proteins are dominant players in the cell in terms of both functionality and biomass, accounting for about half of the total dry mass. They are thus a focus of attention in biological research. With the advent of quantitative proteomics there has been a growing capability to report the copy numbers of proteins in a wide variety of cell types. However, the explicit question of how many protein molecules are in a cell in total often baffles even experienced researchers. Here, we show how it is possible to estimate this value from properties of the cell for which we have quite accurate measurements, and compare it with reported values based on different measurement techniques. Various techniques were used throughout the years to measure protein abundances ranging from colorimetric 1 and spectroscopic 2 to amino acid analysis and radioimmunoassays. Those methods are focused on either total protein or specific protein quantification after proper purification, identification, and calibration. The pioneering efforts to quantify a significant fraction of the proteome using radio isotopic labeling and 2D gels 3 has since given way to a torrent of information from mass spectrometry, which enables comprehensive proteome-wide quantification 4–14.

Discussions of the cell content in terms of absolute values are becoming more common as techniques facilitating such measurements become ever more advanced and the functional implications such as the stochasticity that accompanies the many proteins that exist at very low copy numbers, become exposed 15. Absolute values can also serve as sanity checks on measured reports where, for example, the number of histones in a cell can be constrained by the known DNA length. Similarly, the number of sugar transporters can be constrained, given the growth rate and turnover numbers, as their flux has to suffice to build the cell biomass 16. Ribosomes have been quantified in various studies and can also serve as benchmarks for the absolute copy numbers per cell at a given growth condition. Careful analysis of the absolute values can thus reveal biases in quantification 17. Recent work showed how values from even the most reliable mass spectrometry methods such as iBAQ 18 should be normalized 19 to give reasonable overall cellular concentrations. While experimental measurements are the foundation of our knowledge of biological systems, estimates serve as predictions that are useful in testing where we might be holding misconceptions about cells or about methods that measure their properties.

We begin by deriving an expression for the number of proteins per cell volume. We then use known parameter values to arrive at concrete predictions for the number of proteins per cell volume. These predictions are contrasted with a survey of the reported values in the literature. It is shown how these values differ markedly, and possible reasons for these seeming discrepancies are discussed. Renormalization factors are given that can be used to reinterpret previously reported protein copy numbers to be consistent with the analysis given here.

## Estimating the total number of proteins per cell volume

Protein content scales roughly linearly with cell mass and volume. Given that cell volume can change several fold as a consequence of growth conditions or strain identity, we choose to discuss protein content per unit cell volume. Later, we can multiply by cell volume to arrive at the total protein number per cell under a given growth condition.

**Figure 1:**
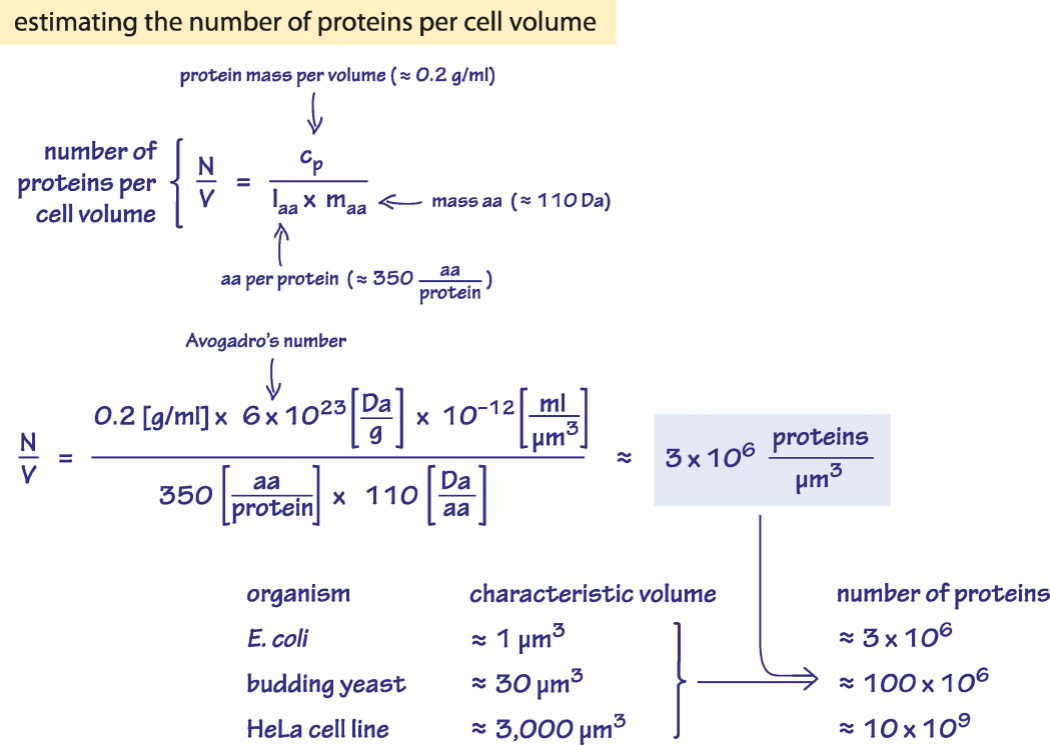
A back of the envelope calculation of the number of proteins per cell volume. Application for selected model organisms based on their characteristic cell volumes is also given. Estimate is based on generic parameter values. For more accurate organism specific values see main text.

Our first method of estimate is shown as a back-of-the-envelope calculation in Fig. 1 using rounded “generic” parameter values. The estimate relies on knowledge of the protein mass per unit volume (denoted by *c*_p_). The units of *c*_p_ are [g protein]/[mL cell volume] and this parameter has been reported for different cell types (see e.g. 20 and also BNID 105938, where here and later in this manuscript pointers for directly assessing values from the literature are given using the BioNumbers database identifiers 21, http://www.bionumbers.hms.harvard.edu). We denote by *l*_aa_ the average length, in amino acids, of a protein, and the average mass of an amino acid by *m*_aa_. The number of proteins per unit volume is equal to: *N*/*V* = *c*_p_/(*l*_aa_ × *m*_aa_).

In *Escherichia coli* and other bacteria, we use an average protein length, *l*_aa_, of 300 aa/protein and in budding yeast, fission yeast, and human cells, we adopt the value of 400 aa/protein (where values are rounded to within about 10–20% accuracy). These average lengths were calculated by weighting the lengths of proteins by their abundance in the cell. This takes into account that high abundance proteins tend to be smaller than low abundance proteins. This calculation used published datasets mentioned in Table 1 (see also 22–23 and BNID 107049, 108983).

**Table 1:** Range of values for the number of proteins per cell based on published studies

Reported proteins per cell	Cell volume (μm^3^)	Inferred proteins per volume (10^6^/μm^3^)	Mismatch from calculation	BNID, Ref, Method
*M. pneumonia*				
0.05 × 10^6^	0.015	3	<2-fold	Kühner et al. 6. Using cryoEM counting of ribosomes for normalization. Volume calculated based on pear shaped 700 nm × 300 nm.
*L. interrogans*				
a(1.0–1.2)×10^6^	0.22	5	<2-fold	Schmidt et al. 7. Summing table SV. Volume given in Maier et al. 36 citing Beck et al. 37.
*E. coli*				
2.36 × 10^6^	0.86	2.7	<2-fold	100088, Neidhardt & Umbarger, EcoSal Ch. 3, 1996, 40 min doubling time, 0.95 pg cell total weight and assuming cell density of 1.1 (used for normalization by Lu 30). Original calculation based on average protein MW = 40 kDa but weighing by abundance gives MW ≈ 30 kDa that will make value higher by a third.
2.3 × 10^6^	0.7	3.3	<2-fold	Arike et al. 18. Volume not specified
a0.1 × 10^6^		N/A	N/A	Taniguchi et al. 32, cover about 1/4 of genome. Growth at 30°C (based on fluorescent protein).
a280 × 10^6^		N/A	N/A	Ishihama et al. 8, they report a massive overestimate in ribosomal protein counts
*B. subtilis*				
a2.3 × 10^6^	1.13	2.0	<2-fold	Maass et al. 33, exponential growth, early stationary, and late stationary, respectively. Only cytosolic proteins with isoelectric point at pH 4–7 quantified.
a1.3 × 10^6^	0.62	2.1	<2-fold	
a1.8 × 10^6^	0.85	2.1	<2-fold	
*S. aureus*				
a0.35 × 10^6^	0.33	1.1	≈3-fold	Maass et al. 33, exponential growth, early stationary, and late stationary, respectively. Only cytosolic proteins with isoelectric point at pH 4–7 quantified.
a0.27 × 10^6^	0.23	1.2	≈3-fold	
a0.26 × 10^6^	0.23	1.1	≈3-fold	
*S. cerevisiae* (haploid)				
50 × 10^6^	≈30–40	1–2	≈2-fold	106198, Futcher et al. 34, based on 1977 paper measuring 4 fg of protein per cell (used for normalization by Lu 30)
a47 × 10^6^				Ghaemmaghami et al. 31, summing up all measured proteins (based on TAP tag).
53 × 10^6^ (30 × 10^6^–80 × 10^6^)				104313, von der Haar 35, the author merged various high throughput measurements.
*S. pombe*				
60.3 × 10^6^	≈100	0.6	≈5-fold	Marguerat et al. 9. Mass Spectrometry, exponential growth.
*M. musculus* (NIH3T3 cells)				
a3 × 10^9^	≈2,000	1.5	<2-fold	Schwanhäusser et al. 10 updated in Nature 2013. SILAC medium. Volume based on BNID 108979.
*H. sapiens* (U2OS)				
a(0.95–1.7) × 10^9^	≈4,000	0.2–0.4	≈10-fold	Beck et al. 11. Range stems from the 11 most highly abundant proteins for which the authors could not calibrate accurately and originally reported as >20 million copies per cell. Assuming 20 million gives the lower value and using the original measured higher values gives the upper bound.
*H. sapiens* (HeLa)				
a2.0 × 10^9^	≈2,000	1	≈3-fold	Nagaraj et al. 12.
a2.3 × 10^9^	≈2,000	1	≈3-fold	Finka and Goloubinoff 19. Analyzing data from Geiger et al. 13.

In some cases the number is inferred from supplementary information and was not reported as such. When cell volume was not reported in the study, literature values under similar conditions were used. Mismatch between values inferred from the literature per unit volume and estimates given in this paper (2–4 million proteins per micron cubed) is calculated.

a Value for total proteins per cell was not explicitly reported and is based on summing the abundance values as reported in the supplementary material across the proteome.

Moving on to the protein concentration in the cells, reports are surprisingly scarce: old measured values for *c*_p_ are 0.24 g/mL for *E. coli* and 0.28 g/mL for budding yeast (BNID 105938, 108879, 108263, see also 24. Values are expected to be similar when the concentration values refer to either the total cell volume and protein complement including membrane associated proteins, or solely to cytoplasmic volume and proteins). Assuming an average amino acid mass of 110 Da – and with some unit conversions – we arrive at:


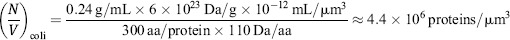


and


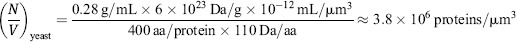


The reader might be wondering about the accuracy of the value of *c*_p_ used. We can derive it based on other, better known, properties: cell density, water content, and protein fraction of dry mass. The total cell density, *d*, is about 1.1 g/mL (BNID 103875, 102239, 106439). The water content, which we denote by w, is in *E. coli* ≈70% and in budding yeast ≈60% by mass (BNID 103689). The protein fraction of the dry mass, *p*, at characteristic exponential growth conditions was measured at ≈55% in *E. coli* and ≈40% in yeast. The relationship between these quantities is: *c*_p_ = *d* × (1 − *w*) × *p*. Plugging in the parameter values:


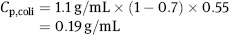


and


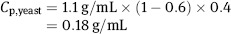


The resulting values are smaller than those quoted above by 20–40%, and they lead to estimates for *E. coli* and budding yeast of, respectively:


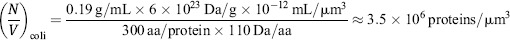


and


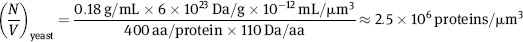


The estimates for the total number of proteins per cell unit volume are calculated based on the overall cell density, the average protein length, and the overall water content. These parameters refer not merely to the cytoplasmic volume but are rather a weighted average that also includes the volumes of organelles (including vacuoles), membranes, the nucleus, and the cell wall. The estimates are therefore representative of the overall average cellular protein content.

For mammalian cells, a value for protein density of *c*_p_ ≈ 0.2 g/mL was reported (BNID 105938, see also 25–26) leading to similar estimates as for budding yeast:


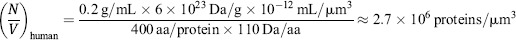


Cell type characteristics can lead to variations, but these are not expected to lead to differences of more than twofold except for very unique cell types. An outlier low protein density of 60 mg/mL has been mentioned 19 yet this is not considered a representative value in the literature. The calculation here does not account for secreted proteins, which are expected to be a small fraction in most cell lines (some immune cells might be notable exceptions).

We can now use characteristic volumes to reach the number of proteins per cell. For an *E. coli* cell of 1 μm^3^ volume (average values often vary between 0.5 and 2 μm^3^ depending on growth rate and conditions), the estimates give a range of 3–4 million proteins per cell. For a haploid budding yeast cell of characteristic volume 40 μm^3^, the two estimates give a range of 100–150 million proteins per cell. Applying the estimated protein densities to mammalian cell volumes yields a value of about 10^10^ proteins per cell for cell lines with characteristic volumes of 2,000–4,000 μm^3^. Yet because cell volume can change several fold under different growth conditions, it is usually much more accurate to use values per unit volume rather than a total protein count per cell.

## Comparison to values reported in the literature and discussion of possible sources of mismatch

How do these values compare to previous reports in the literature? Table 1 shows a compilation of values based on published proteome-wide studies. Notably, in many cases a total sum over all proteins was not reported and was inferred for our purposes by summing all measured abundances. While many of the total sums are within twofold of the estimates above, some values – most notably for eukaryotic cells including yeast and mammalian cells – are lower than predicted by as much as a factor of 5–10. This is beyond the uncertainty that can be explained by the ranges of the parameters that went into the estimates. What can possibly explain the marked differences between some reported values and the estimates above?

In mass spectrometry measurements, a fraction of the proteome is not reported because the measured values are below the reliable detection limit. Even though the number of such genes can be significant (measuring in thousands), current sensitivity is so good that the contribution to the overall sum is expected to be negligible, amounting to much less than 20% of the overall count. The small quantitative effect of the proteins that are below the detection limit is in line with the fact that the top 1,000 most highly expressed proteins in a cell make up over 80% of the proteome mass as calculated by the author from the data published in papers referred to in Table 1. In most cells, the fraction of the proteome covered by the top 1,000 proteins is actually over 90% of the protein copies (as well as amino acids), see also 12,14. The fraction of unmeasured protein thus cannot explain the differences observed between the reported values and the estimate prediction.

Vacuoles are a significant constituent of eukaryotic cells. In some plant cells, they can often occupy 80–90% of the cell volume (BNID 103442, 104368) and tend to have a lower protein concentration. Can this explain the seeming discrepancy observed here for yeast and mammalian cells? For budding yeast, the vacuole can be >50% of the cell volume under conditions of stress; however, it is generally much smaller – 10–20% of cell volume – under exponential growth with glucose. Moreover, the measured parameter of protein concentration per unit volume, *c*_p_, which was used in the estimates above applies to intact cells, including any vacuole fraction. If there is, indeed, within the vacuole a lower abundance of proteins than in the other parts of the cell, the concentration should be even higher in the other parts of the cell in order to achieve the overall measured concentration. This should not have an effect on the overall estimate for the number of proteins per cell unit volume. Finally, in mammalian cells vacuoles are much more rare. It is therefore hard to see how vacuoles explain the observed mismatches between some reported values for the overall number of proteins per unit volume and the estimates provided here.

In label-based approaches, such as fluorescent tagging, different groups of proteins can be resistant to tagging, and thus not quantified. Ribosomal proteins, which can account for over a fifth of the proteome under fast growth rates 27, often fall into this category. It is also known that there can be major biases in measuring non-cytoplasmic proteins. Mass spectrometry can be limited in measuring membrane-associated proteins. For example, the highly abundant (BNID 100082, 28) hydrophobic lipoprotein Lpp connected to the outer membrane and peptidoglycan of *E. coli* was not observed in some mass spectrometry studies. The higher number of protein modifications in eukaryotes can reduce the measured protein abundances. Though these technical biases can cause underestimates of the cell protein count, it is difficult to see how this can account for the major part of the 5–10-fold mismatch shown in Table 1 for fission yeast and a U2OS human cell line. A more “global” scaling factor is probably required based on technical issues in the quantification (or in the calculation above). Such a global factor can be loss of material during cell lysis, tryptic digest, and preparation for mass spectrometry, or any other step that is not adequately corrected via standards. Another explanation can be an erroneous factor applied somewhere in the very elaborate set of calculations used to derive absolute quantification values.

## The need to measure cell volumes and key physiological parameters

The values of characteristic cell parameters used here for the calculations of protein content per cell dry mass or per cell volume are mostly based on physiological studies from decades ago. Probably the least robust value is that of the protein content per cell unit volume (usually reported in mg of protein per mL of cell volume). It is worthwhile revisiting these values with the most modern techniques. Yet, it is hard to imagine that a modernized version of the physiology experiments for fission yeast and human cell lines will explain the 5–10-fold difference seen between simple estimates and measured values. If – and that would be surprising – newer measurements will show the values to be so significantly different from those found in previous measurements, this would be an important step forward in our understanding of cell composition.

Another parameter that is suspected is the value used for cell sizes. Measurements of the average cell volume for a population of cells can have accuracy of better than 10%, for example by using the standard Coulter cell counter. Yet in many of the studies on quantitative proteomics there is no concurrent measurement of cell volume reported. In some cases, there is no value for the cell volume at all and in other cases inference is made based on reported measurements of cell diameters. In these cases, it is possible that the cells whose proteomes were quantified were somehow much smaller than those previously reported in the literature, and whose volume served as the basis for our estimates of the mismatch from the calculations as shown in Table 1. It is known that cell volume can change several fold in response to subtle growth conditions; an example is HeLa cell volume as a function of time since splitting and re-plating 29. However, we do not see a reason for which such biases in cell size would be consistently in the direction required to explain the mismatch between the reports and the estimate given here. Moreover, this explanation can also be tested by looking at copy numbers of proteins that should not depend on cell size, e.g. histones. A major take-home message of the current analysis is the crucial importance of measuring cell volumes (using the Coulter cell counter, microscopy, or some other method) in any future proteomics study that seeks to report absolute protein copy numbers.

## Conclusions and outlook

Mass spectrometry is the most common method for quantification, and is the method used in the studies showing the most marked mismatches with the estimates. The source of the mismatch from the calculation presented here is unclear at this time. Some methods of analysis result in measured values close to the estimate, whereas others deviate significantly. Hence, it seems that a careful comparative analysis of the calibration steps, as well as a direct measurement of the protein mass per cell volume, will clarify the underlying reasons for the mismatch. It may take time – hopefully not too long – to reveal the sources of the seeming discrepancies and report updated values or to re-measure and revise literature reported cell composition properties used as assumptions in the estimate. In the meantime, the information in Table 1 can be used to rescale reported values of protein abundance to fit the estimates for the overall cell content. The multiplicative scaling factor needed is the ratio between the estimated 2–4 × 10^6^ protein/μm^3^ and the value calculated in Table 1, protein per unit volume column. For example, the fission yeast protein counts can be multiplied ≈5-fold (between 2/0.6 and 4/0.6). The need for such “normalization” was recently pointed out 19 after noting that summing protein abundances results in nonrealistic overall protein concentrations. It is advised that researchers performing future proteome-wide quantitative studies compare their summed abundances to the estimates given here as a crude sanity check. Such a simple analysis will ensure consistency across studies of the values attained through the impressive technical achievement of proteome-wide abundance datasets.

Novel high-throughput methods in proteomics hold the promise for a detailed census of the proteome. This study indicates that there remain important challenges for careful calibration in order to achieve definitive answers for those interested in quantitatively mapping the cell's contents.

## Abbreviations

BNID: BioNumbers website IDentification number
iBAQ: intensity based absolute quantification
aa: amino acids
MW: molecular weight
kDa: kilodaltons

## References

[b1] Sapan CV, Lundblad RL, Price NC (1999). Colorimetric protein assay techniques. Biotechnol Appl Biochem.

[b2] Aitken A, Learmonth M (2002). Protein determination by UV absorption. Notes.

[b3] Pedersen S, Bloch PL, Reeh S, Neidhardt FC (1978). Patterns of protein synthesis in *E. coli*: a catalog of the amount of 140 individual proteins at different growth rates. Cell.

[b4] Cox J, Mann M (2011). Quantitative, high-resolution proteomics for data-driven systems biology. Annu Rev Biochem.

[b5] Aebersold R, Mann M (2003). Mass spectrometry-based proteomics. Nature.

[b6] Kühner S, Van Noort V, Betts MJ, Leo-Macias A (2009). Proteome organization in a genome-reduced bacterium. Science (New York, NY).

[b7] Schmidt A, Beck M, Malmström J, Lam H (2011). Absolute quantification of microbial proteomes at different states by directed mass spectrometry. Mol Syst Biol.

[b8] Ishihama Y, Schmidt T, Rappsilber J, Mann M (2008). Protein abundance profiling of the *Escherichia coli* cytosol. BMC Genomics.

[b9] Marguerat S, Schmidt A, Codlin S, Chen W (2012). Quantitative analysis of fission yeast transcriptomes and proteomes in proliferating and quiescent cells. Cell.

[b10] Schwanhäusser B, Busse D, Li N, Dittmar G (2011). Global quantification of mammalian gene expression control. Nature.

[b11] Beck M, Schmidt A, Malmstroem J, Claassen M (2011). The quantitative proteome of a human cell line. Mol Syst Biol.

[b12] Nagaraj N, Wisniewski JR, Geiger T, Cox J (2011). Deep proteome and transcriptome mapping of a human cancer cell line. Mol Syst Biol.

[b13] Geiger T, Wehner A, Schaab C, Cox J (2012). Comparative proteomic analysis of eleven common cell lines reveals ubiquitous but varying expression of most proteins. Mol Cell Proteomics.

[b14] Geiger T, Velic A, Macek B, Lundberg E (2013). Initial quantitative proteomic map of twenty-eight mouse tissues using the SILAC mouse. Mol Cell Proteomics.

[b15] Eldar A, Elowitz MB (2010). Functional roles for noise in genetic circuits. Nature.

[b16] Phillips R, Milo R (2009). A feeling for the numbers in biology. Proc Natl Acad Sci USA.

[b17] Schwanhäusser B, Busse D, Li N, Dittmar G (2013). Corrigendum: global quantification of mammalian gene expression control. Nature.

[b18] Arike L, Valgepea K, Peil L, Nahku R (2012). Comparison and applications of label-free absolute proteome quantification methods on *Escherichia coli*. J Proteomics.

[b19] Finka A, Goloubinoff P (2013). Proteomic data from human cell cultures refine mechanisms of chaperone-mediated protein homeostasis. Cell Stress Chaperones.

[b20] Albe KR, Butler MH, Wright BE (1990). Cellular concentrations of enzymes and their substrates. J Theor Biol.

[b21] Milo R, Jorgensen P, Moran U, Weber G (2010). BioNumbers—the database of key numbers in molecular and cell biology. Nucl Acids Res.

[b22] Brocchieri L, Karlin S (2005). Protein length in eukaryotic and prokaryotic proteomes. Nucl Acids Res.

[b23] Zhang J (2000). Protein-length distributions for the three domains of life. Trends Genet.

[b24] Zimmerman SB, Trach SO (1991). Estimation of macromolecule concentrations and excluded volume effects for the cytoplasm of *Escherichia coli*. J Mol Biol.

[b25] Ellis RJ (2001). Macromolecular crowding: an important but neglected aspect of the intracellular environment. Curr Opin Struct Biol.

[b26] Wang Z, Shen W, Kotler DP, Heshka S (2003). Total body protein: a new cellular level mass and distribution prediction model. Am J Clin Nutr.

[b27] Dennis PP, Bremer H (1974). Macromolecular composition during steady-state growth of *Escherichia coli* B-r. J Bacteriol.

[b28] Inoyye S, Takeishi K, Lee N, DeMartini M (1976). Lipoprotein from the outer membrane of *Escherichia coli*: purification, paracrystallization, and some properties of its free form. J Bacteriol.

[b29] Luciani AM, Rosi A, Matarrese P, Arancia G (2001). Changes in cell volume and internal sodium concentration in HeLa cells during exponential growth and following lonidamine treatment. Eur J Cell Biol.

[b30] Lu P, Vogel C, Wang R, Yao X (2007). Absolute protein expression profiling estimates the relative contributions of transcriptional and translational regulation. Nat Biotechnol.

[b31] Ghaemmaghami S, Huh W-K, Bower K, Howson RW (2003). Global analysis of protein expression in yeast. Nature.

[b32] Taniguchi Y, Choi PJ, Li G, Chen H (2010). Quantifying *E. coli* proteome and transcriptome with single-molecule sensitivity in single cells. Science.

[b33] Maass S, Sievers S, Zühlke D, Kuzinski J (2011). Efficient, global-scale quantification of absolute protein amounts by integration of targeted mass spectrometry and two-dimensional gel-based proteomics. Anal Chem.

[b34] Futcher B, Latter GI, Monardo P, McLaughlin CS (1999). A sampling of the yeast proteome. Mol Cell Biol.

[b35] Von der Haar T (2008). A quantitative estimation of the global translational activity in logarithmically growing yeast cells. BMC Syst Biol.

[b36] Maier T, Schmidt A, Güell M, Kühner S (2011). Quantification of mRNA and protein and integration with protein turnover in a bacterium. Mol Sys Biol.

[b37] Beck M, Malmström JA, Lange V, Schmidt A (2009). Visual proteomics of the human pathogen *Leptospira interrogans*. Nat Methods.

